# Influence of Social Support on Teachers' Attitudes Toward Inclusive Education

**DOI:** 10.3389/fpsyg.2021.736535

**Published:** 2021-09-30

**Authors:** Caroline Desombre, Marine Delaval, Mickaël Jury

**Affiliations:** ^1^Univ. Lille, ULR 4072—PSITEC—Psychologie: Interactions, Temps, Emotions, Cognition, Lille, France; ^2^Activité, Connaissance, Connaissance, Transmission, Éducation (ACTe), Universite Clermont Auvergne, Clermont-Ferrand, France

**Keywords:** attitude toward inclusive education, social support, teachers, students with SEN, school

## Abstract

Inclusive education is at the heart of educational policy world-wide. Teachers' attitudes toward inclusive education, which are often associated with the success of the policy, have been studied extensively. Various factors related to teachers, students with special educational needs (SEN) and different specific contexts have been identified. In the current study, we explored the influence of social support on teachers' attitudes toward inclusive education. In a pilot study implying teachers, we replicated, in the French context, previous results showing a correlational link between social support and attitudes toward inclusion. Specifically, we showed that the more social support they perceived with regard to their attempts to include students with SEN, the more positive the teachers' attitudes toward inclusive education. In an experiment involving 314 teachers we then explored the causal link between these variables. Results showed that highlighting the support teachers receive improves their attitudes in comparison with highlighting a lack of support or a control condition in which support is not mentioned. These studies show the importance of supporting inclusive education in the schools. This support can be provided in different ways (emotional, informational, instrumental, etc.) and by different actors (colleagues, supervisors).

## Introduction

The Salamanca Statement on Principles, Policy, and Practice in Special Needs Education (UNESCO, 1994) has given rise to a range of changes regarding the schooling of students with disability. All over the world, many laws and decrees have been adopted in recent years to promote and implement inclusive education (Schwab, 2020). To this end, in France for example, the Ministry of Education has recently proposed a law ensuring that all pre-service and in-service teachers in the French school system would receive specific training regarding the inclusion of students with special educational needs (SEN).

Despite a growing support (e.g., a 7.6% increase within the inclusive education budget last year in France), many obstacles to a fully inclusive education remain over the world (see for example Westwood and Graham, 2003) and in particular in the French system (Berzin et al., 2020). Numerous studies have been conducted to identify these barriers, particularly among teachers. One of the most studied variables is teachers' attitudes toward inclusive education. It has been hypothesized that the more reserved the teachers are regarding the overall inclusive education policy, the less they will personally endorse inclusive pedagogical procedures (MacFarlane and Woolfson, 2013; Sharma and Sokal, 2016). Among the factors known to influence these attitudes (for reviews, see Avramidis and Norwich, 2002; de Boer et al., 2011), their perceived social support has been of particular interest. For example, Hind et al. (2019) recently showed that teachers do not always feel supported in their attempts to implement the inclusion policy and that this perceived lack of support is associated with negative attitudes. The purpose of the present study was to go beyond the correlational nature of these findings and experimentally investigate how teachers' perception of the support they receive could influence their attitudes in order to draw causal conclusion.

### Attitudes Toward Inclusive Education

Attitudes were defined as “a psychological tendency that is expressed by evaluating a particular entity with some degree of favor or disfavor” (Eagly and Chaiken, 1993, p. 1). Teachers' attitudes toward inclusive education specifically refer to their beliefs, feelings, and intentions toward an inclusive policy. The scientific literature tends to show that these attitudes are somewhat positive and that the more they are, the more teachers' are willing to include students with SEN in mainstream education (Wertheim and Leyser, 2002; MacFarlane and Woolfson, 2013; Wilson et al., 2016; Hind et al., 2019), the more welcoming is the classroom learning environment (Monsen et al., 2014) and the more students with SEN feel socially integrated (Ben-Yehuda et al., 2010; Heyder et al., 2020). However, these attitudes are also ambiguous (Scruggs and Mastropieri, 1996; Krischler et al., 2018; for a review see van Steen and Wilson, 2020) since teachers generally support the philosophy of inclusive education but are reluctant to implement it in their own classrooms (Alghazo and Naggar Gaad, 2004; Hwang and Evans, 2011).

Many studies have been conducted to identify variables related to teachers' attitudes toward inclusive education. The most commonly targeted categories are teacher-related, student-related, and environment-related variables. Teacher-related ones refer to teachers' experience (Glaubman and Lifshitz, 2001; Alghazo and Naggar Gaad, 2004), gender (Alghazo and Naggar Gaad, 2004; Parasuram, 2006), training (Tournaki and Samuels, 2016; Lautenbach and Heyder, 2019), self-efficacy (Pit-ten Cate et al., 2018), values (Perrin et al., 2021), or position (McHatton and Parker, 2013; Desombre et al., 2019). Student-related variables identified as having an influence on teachers' attitudes include the type of disabilities/difficulties the students exhibit (Krischler and Pit-ten Cate, 2019; Jury et al., 2021a,b), and the extent to which instructional practices should be modified to meet students' special needs (Center and Ward, 1987). For example, teachers expect students with emotional and behavioral difficulties as well as those with profound and complex learning difficulties to be the most difficult to include (Avramidis and Norwich, 2002).

Finally, environment-related variables refer to the country's educational policies (Savolainen et al., 2012), cultural differences among teachers (Leyser et al., 1994), educators' understanding of inclusive education policy (Krischler and Pit-ten Cate, 2019), or school resources and support available for teachers (Minke et al., 1996; Avramidis and Norwich, 2002). To achieve inclusive education, teachers need to be confident both in their personal competences to meet the special needs of their students and in the collective competences to move toward a more inclusive school (Curchod-Ruedi et al., 2013). Collective competence refers to the support a teacher can receive in his or her classroom. According to Brackenreed (2008), one of the most important sources of stress for teachers who include in their classrooms students with SEN is the lack of adequate support, which gives rise to the feeling of being alone to deal with the situation. This may be true even for teachers who agree in general with the policy of inclusive education.

### Social Support and Attitudes Toward Inclusive Education

Social support has been the subject of growing scientific interest since the 1970s. Despite this expansion, this construct has been defined in many ways without reaching consensus among investigators (Pearson, 1986). Indeed, various specific types of social support have been identified with no agreement regarding neither their number nor types (Barling et al., 1988). At school, and for the present study, three types of social support can be identified (Langford et al., 1997). More precisely, teachers could receive instrumental support, which refers to providing assistance such as material goods and services or sharing of tasks or responsibilities, informational support, which relates to providing suggestions, advice, feedback, and problem-solving assistance, and emotional support referring to exchanges of empathy, expressions of caring, concern, affection, or encouragement.

Studies conducted in different contexts (professional, health, social networks) have shown that social support helps to reduce depression (Hays et al., 1990) and physical distress (Leserman et al., 1999) and bolsters psychological well-being (Hays et al., 1990). It should be noted that the concept of social support includes both perceived and actual social supports (McDowell and Serovich, 2007). Perceived social support appears to have more effect on health than actual availability of social supports (McDowell and Serovich, 2007).

Regarding inclusive education, social support can come from a variety of sources (Villa et al., 1996; Werts et al., 1996): peers, administrators, superiors, or resources (training, support from a team of experts, and support in the classroom). The link between these various sources of support and teachers' attitudes toward inclusive education has been identified in several studies. For example, peers support was identified as being an important influence on teachers' positive attitudes toward inclusive education policies (Boyle et al., 2012). In the same way, Rodríguez et al. (2012) showed that being part of a support network improved special educators' attitude toward the inclusion of students with autism spectrum disorders (ASD). Training has also been found to support teachers in the implementation of inclusive education and improve their attitudes toward inclusive education (Tournaki and Samuels, 2016; Pit-ten Cate et al., 2018; Li et al., 2019).

Although aimed at similar goals, the foregoing studies are intrinsically limited. Indeed, they either focus on specific SENs (i.e., students with ASD, Rodríguez et al., 2012; Li et al., 2019) or on specific teacher profiles (Tournaki and Samuels, 2016; Li et al., 2019). In addition, these studies focus on only one source of support, which suggests a need to study the role of social supports in a more general way. Finally, the methods used in these studies are mainly qualitative or correlational. Thus, using an experimental design would allow a sounder understanding of how social support predict teachers' attitudes toward inclusive education.

In the present study, we attempted to better understand the influence of social support on teachers' attitudes toward inclusive education. To do so, we first aimed to replicate in a pilot study the correlational link between these two constructs in the French context. Indeed, in addition to the importance of replication in educational and social psychology (Open Science Collaboration, 2017), the cultural differences in the inclusive education policy and corresponding teachers' attitudes (see for example, Moberg et al., 2020) imply that previous results from other countries could not be taken for granted. Then, we proposed to test the causal link between social support and teachers' attitudes within a controlled experiment. Based on the literature cited above (Minke et al., 1996; Avramidis and Norwich, 2002), we hypothesized that inducing a positive social support would enhance teachers' attitudes toward inclusive education.

In this study no data were excluded, all data were collected before any analyses were conducted, and all variables that were analyzed are reported. All material and data regarding this project can be accessed at: https://osf.io/6jgex/.

## Pilot Study

### Participants

Two hundred and sixty-two teachers (163 general education teachers and 98 special educators, 1 missing value) participated in this pilot study. The sample included 203 females, 58 males and one who did not identify as either male or female. Mean age was 41.75 (*SD* = 9.73); mean teaching experience of 17.51 years (*SD* = 10.27).

### Materials

#### Attitudes Toward Inclusive Education

Participants completed a tool created by Mahat (2008), the Multidimensional Attitudes toward Inclusive Education Scale (MATIES). Among the range of available scales measuring teachers' attitudes, the MATIES has been identified as having the most adequate psychometric properties to measure this construct (Ewing et al., 2018). This 18-item scale assesses teachers' attitudes toward inclusive education (e.g., “I believe that an inclusive school is one that permits academic progression of all students regardless of their ability,” “I get irritated when I am unable to understand students with a disability.”—reversed item) on a five-point scale from 1, “Totally disagree,” to 5, “Totally agree.” In our sample reliability was satisfactory (α = 0.85) and mean score is equal to 4.06 (*SD* = 0.54).

#### Perceived Social Support

To our knowledge, there is no available tool in the literature to measure teachers perceived social support regarding inclusive education. Therefore, participants were asked to complete a seven-item scale adapted from the one developed by Lauzier et al. (2015) which originally seeks to measure students' social support. This new scale, using the five-point scale described above, should allow us to measure teachers' perceived social support regarding the inclusion of students with SEN [e.g., for informational support, “I have sufficient support to ensure the successful inclusion of students with Special Educational Needs.,” for instrumental support, “I have the resources available when I have questions about students with Special Educational Needs.,” for emotional support, “I am alone to face the difficulties of students with special educational needs.” (Reversed item)]. For our sample reliability was satisfactory (α = 0.91) and mean score was equal to 2.81 (*SD* = 1.09).

### Procedure

During the spring semester of the 2018–2019 school year, potential volunteers were invited by email to participate in an online study. The email was sent to and by academic authorities, so the investigators did not know the potential participants. This email informed them about the study's main topic (i.e., inclusive education) as well as its procedure (i.e., an online questionnaire that should be answered in 10 min). In addition, they learned that their participation was voluntary, that they could quit the study at any time without consequences and that they would not receive any financial compensation. Upon consenting, participants were asked to fill in the questionnaires. At the end, they were debriefed regarding the goal of the study.

### Results and Discussion

To test the hypothesis that the more teachers perceive that they receive social support, the more positive their attitudes will be, a regression analysis has been conducted. As anticipated, results confirmed that the more teachers perceived social support regarding the inclusion of students with SEN, the more positive their attitudes toward inclusive education, *B* = 0.23, *t*_(260)_ = 8.38, *p* < 0.001, η_*p*_^2^ = 0.21, 95% CI [0.17; 0.28]^1^.

Results from this pilot study confirmed the hypothesis in the French context. However, as mentioned above, the study's correlational nature limits the extent to which causal conclusions could be drawn regarding the impact of social support on teachers' attitudes. Consequently, a pre-registered experiment was conducted to test the hypothesis assuming that a positive social support could improve teachers' attitudes in comparison with a negative social support or a control condition in which social support would not be mentioned.

## Main Study

### Participants

As indicated in the preregistered form (AsPredicted#34492^1^), an a priori power analysis performed with G^*^power 3.1 (Faul et al., 2007) revealed that 152 participants would be needed to detect a small effect (*f* = 0.22) with targeted power of 0.80. This was the minimum targeted threshold. Ultimately, 314 pre-service and in-service teachers participated in the data collection. The sample included 240 females, 72 males and two who did not identify as either male or female. For the whole sample, the mean age was 34.29 years (*SD* = 11.07) and the mean teaching experience was 8.97 years (*SD* = 10.66). Among the participants, 124 were general education teachers, 50 were special educators and 140 were pre-service general education teachers. No information about their experience with students with SEN was collected.

### Material

#### Manipulation of the Social Support

Participants were randomly assigned to one of three experimental conditions. They were then asked to read a fake journal article, written especially for this study by three experts of the field, containing approximately 350 words that highlighted either the positive social support teachers received from the ministry regarding inclusive education, or the lack of such support, or a control condition in which social support was not mentioned. It is worth noting that even if disentangling the specific effects of the distinct types of social support (instrumental, informational, and emotional) was outside the scope of the present paper, they were considered within the positive and negative social support condition.

More precisely, if the article was about inclusive education with a common introduction, it differed according to the experimental condition. Within the positive social support condition (*n* = 103), participants could read about the various support measures they received such as teacher coaching (i.e., informational support), training and the availability of resources to facilitate pedagogical accommodations (i.e., instrumental support) in order to emphasize the fact that teachers feel supported (e.g., “I feel more and more supported by the institution and by my colleagues when trying to better include my students,” emotional support). Contrarily, in the negative support condition (*n* = 110), the article mentioned the lack of support on the same dimensions (instrumental, informational, and emotional) in order to emphasize the fact that teachers do not feel supported. Despite their disparities, these two articles shared 90% of the content. Finally, in the control condition (no mention of supports, *n* = 101), the article simply describes the possible schooling pathways for students with SEN and the decision-making structures for student orientation. It should be noted that these three articles and their translation in English are available online (see link above).

#### Attitudes Toward Inclusive Education

Participants completed the same scale as the one use in the pilot study (Mahat, 2008). For the present sample reliability was satisfactory (α = 0.87) and the mean score is equal to 3.91 (*SD* = 0.62).

### Procedure

During the fall semester of the 2019–2020 school year, potential volunteers were invited by email to participate in an online study. As in the pilot study, this email informed them about the study's main topic (i.e., inclusive education) as well as its procedure (i.e., a 10 min online questionnaire). As previously, participants read that their participation was voluntary, that they could quit the study at any time without consequences and that they would not receive any financial compensation. Upon giving consent, participants were asked to read the instructions and fill in the attitudes measure. At the end, they were debriefed regarding the goal of the study.

### Results

As indicated in the pre-registration form, and due to the specificity of our hypothesis, we have decided to conduct contrast analyses (see Brauer and McClelland, 2005). Indeed, “precise conclusions can be obtained from contrast analysis because a contrast expresses a specific question about the pattern of results of an Anova” (see Abdi and Williams, 2010, p. 253). Therefore, the regression model tested two contrasts: the contrast of interest compared the positive support condition (coded +2) to the control condition and the negative support condition (coded −1 each), assuming that participants in the control condition perceived low support. The orthogonal contrast compared the control condition (coded +1) to the negative support condition (coded −1, the positive support condition being coded 0). To fully support our hypothesis, the contrast of interest should be significant while the orthogonal one should not. Preliminary analysis indicated that three participants had studentized residuals below −3.29 and thus were cause for concern and deleted from the present sample (Field et al., 2012)^2^.The regression analysis revealed that the contrast of interest is significant, *B* = 0.07, *t*_(311)_ = 2.81, *p* = 0.005, η_*p*_^2^ = 0.02, 95% CI [0.02; 0.11], indicating that participants in the positive support condition (*M* = 4.05, *SD* = 0.57) express more positive attitudes than participants in the control condition (*M* = 3.97, *SD* = 0.57) or the negative support condition (*M* = 3.73, *SD* = 0.66). However, it should be noted that the orthogonal contrast is also significant, *B* =0.12, *t*_(311)_ = 2.92, *p* = 0.004, η_*p*_^2^ = 0.02, 95% CI [0.03; 0.20], indicating that the contrast of interest did not fully explain the variance and that participants in the negative support condition tend to express more negative attitudes than participants in the control condition. In other words, our hypothesis is only partially supported by our data.

## Discussion

At an international level, a growing number of changes were made to support the development of inclusive education for students with SENs (Curchod-Ruedi et al., 2013). These changes refer, for examples, to the increase in the number of training courses in the field of inclusive education for teachers as well as accommodations for students with SEN (e.g., recruiting teaching assistants). Despite these efforts, inclusive education remains a contentious issue (Plaisance, 2010) with many obstacles remaining. A wide range of studies have identified teachers' attitudes toward inclusive education as being an important factor in the implementation of inclusive education and it remains a point of interest to understand which variables influence these attitudes. Among them, the present paper chose to focus on a contextual characteristic, namely, social support. Teachers often point to the lack of financial and institutional support regarding the implementation of inclusive education (Hind et al., 2019). The goal of the present study was to test, in the French context, the correlational link between social support and teachers' attitudes toward inclusive education and the causal link between these two constructs. Based on previous results (Minke et al., 1996; Avramidis and Norwich, 2002), we hypothesized that the more teachers feel supported, the more they positive their attitudes toward inclusive education would be. The findings are consistent with this prediction. The pilot study showed that the higher the perceived social support, the more positive teachers' attitudes. In the main study, we showed that highlighting the social support that teachers receive improves their attitudes in comparison with highlighting a lack of social support or a neutral control condition in which social support was not mentioned. This result suggests that there is a causal link between social support and attitudes toward inclusive education, indicating that enhancing the former could incline teachers to be more inclusive of students with special needs and therefore being more willing to change their practices.

However, these results should be read with caution since the orthogonal contrast in our data analysis was also significant, suggesting that our hypothesis did not fully account for teachers' attitudes and that the perception of negative social support should also be considered. In other words, if making salient the social support could improve teachers' attitudes, it seems that highlighting the lack of social support could lessen these ones. If this result is not contradictory with our hypothesis, it was unexpected since we assume that teachers in the control condition would have necessarily perceived low social support (due to the previous literature) and would not differ from their peers in the negative social support condition. It might be possible that the given information regarding student guidance systems or content of different laws in the former condition have somewhat improved their perceptions of social support, despite our goal. A replication with a more neutral control condition would be necessary.

In addition to this limitation, we should acknowledge that different kinds of social support (i.e., instrumental, informational, and emotional) were all manipulated at the same time within the experimental conditions which prevent us to identify the main source of influence. If this choice was assumed in the present study, future studies could examine if the link between social support and teachers' attitudes could vary depending on the source of social support. Considering that teachers often point to the lack of training (Hind et al., 2019) and thus the lack of informational support, it is possible that this kind of support influences more strongly their attitudes. Future studies could explore this hypothesis. Finally, the finding should also be interpreted with caution since the causal link was tested in a single study with a newly developed experimental material (i.e., the fake journal articles). A replication of these results with these articles and a measured of the associated perceived social support (with a psychometrically validated tool) would definitively strengthen these results.

Notwithstanding these limitations, we believe that these findings have theoretical and practical implications. From a theoretical perspective, identifying the causal link between social support and attitudes is an important contribution. Indeed, studies conducted on teachers' attitudes toward inclusion are mostly correlational which prevent to make causal conclusions and think through leverages. However, by knowing the causal role of social support on teachers' attitudes, these results allow us to consider new ways of accompanying teachers to improve their attitudes and, consequently, the implementation of inclusive education. More precisely, it invites practitioners and decision makers to seriously consider teachers' claim for social support (Rodríguez et al., 2012) and invest on a daily basis to strengthen the instrumental, the informational and the emotional support they receive from their hierarchy (i.e., school principal or) and their colleagues (e.g., the special education teachers, Snyder, 1999). By doing so, our study suggests that it would influence teachers' attitudes toward inclusive education, and consequently their willingness to use inclusive pedagogical practices (MacFarlane and Woolfson, 2013; Sharma and Sokal, 2016; Wilson et al., 2016) as well as the social integration of students with SEN (Heyder et al., 2020). In other words, there is thus a great potential value in understanding the factors that can influence these attitudes and hence allow every student to fully enjoy and benefit from their educational rights.

## Data Availability Statement

The datasets presented in this study can be found in online repositories. The names of the repository/repositories and accession number(s) can be found below: https://osf.io/6jgex/.

## Ethics Statement

Ethical review and approval was not required for the study on human participants in accordance with the local legislation and institutional requirements. The patients/participants provided their written informed consent to participate in this study.

## Author Contributions

CD, MD, and MJ conceived and designed the study and collected the data. MJ analyzed the data. CD drafted the manuscript and MD and MJ provided critical revisions. All authors approved the final version of the manuscript for submission.

## Funding

This study was funded by the Institut National Supérieur du Professorat et de l'Éducation de l'académie de Lille - Hauts-de-France and by the French State in the context of the action “Territories of educational innovation” of the Program of investments of the future, operated by the Caisse des Dépôts (PIA3 - 100% IDT).

## Conflict of Interest

The authors declare that the research was conducted in the absence of any commercial or financial relationships that could be construed as a potential conflict of interest.

## Publisher's Note

All claims expressed in this article are solely those of the authors and do not necessarily represent those of their affiliated organizations, or those of the publisher, the editors and the reviewers. Any product that may be evaluated in this article, or claim that may be made by its manufacturer, is not guaranteed or endorsed by the publisher.

## Footnotes

^1^The pre-registered form regarding this project can be accessed at: http://aspredicted.org/blind.php?x=ze3ds2

^2^Results were identical with or without these participants.
